# Gene expression profiling of intestinal regeneration in the sea cucumber

**DOI:** 10.1186/1471-2164-10-262

**Published:** 2009-06-08

**Authors:** Pablo A Ortiz-Pineda, Francisco Ramírez-Gómez, Judit Pérez-Ortiz, Sebastián González-Díaz, Francisco Santiago-De Jesús, Josue Hernández-Pasos, Cristina Del Valle-Avila, Carmencita Rojas-Cartagena, Edna C Suárez-Castillo, Karen Tossas, Ana T Méndez-Merced, José L Roig-López, Humberto Ortiz-Zuazaga, José E García-Arrarás

**Affiliations:** 1 University of Puerto Rico, Rio Piedras, Department of Biology, San Juan, PR, USA; 2 Yale University, Department of Molecular, Cellular and Developmental Biology, New Haven, CT, USA; 3 Universidad del Este- SUAGM, School of Science and Technology, Carolina, PR, USA; 4 University of Puerto Rico, HPCf, High Performance Computing facility, San Juan, PR, USA

## Abstract

**Background:**

Among deuterostomes, the regenerative potential is maximally expressed in echinoderms, animals that can quickly replace most injured organs. In particular, sea cucumbers are excellent models for studying organ regeneration since they regenerate their digestive tract after evisceration. However, echinoderms have been sidelined in modern regeneration studies partially because of the lack of genome-wide profiling approaches afforded by modern genomic tools.

For the last decade, our laboratory has been using the sea cucumber *Holothuria glaberrima *to dissect the cellular and molecular events that allow for such amazing regenerative processes. We have already established an EST database obtained from cDNA libraries of normal and regenerating intestine at two different regeneration stages. This database now has over 7000 sequences.

**Results:**

In the present work we used a custom-made microchip from Agilent with 60-mer probes for these ESTs, to determine the gene expression profile during intestinal regeneration. Here we compared the expression profile of animals at three different intestinal regeneration stages (3-, 7- and 14-days post evisceration) against the profile from normal (uneviscerated) intestines. The number of differentially expressed probes ranged from 70% at p < 0.05 to 39% at p < 0.001. Clustering analyses show specific profiles of expression for early (first week) and late (second week) regeneration stages. We used semiquantitative reverse transcriptase polymerase chain reaction (RT-PCR) to validate the expression profile of fifteen microarray detected differentially expressed genes which resulted in over 86% concordance between both techniques. Most of the differentially expressed ESTs showed no clear similarity to sequences in the databases and might represent novel genes associated with regeneration. However, other ESTs were similar to genes known to be involved in regeneration-related processes, wound healing, cell proliferation, differentiation, morphological plasticity, cell survival, stress response, immune challenge, and neoplastic transformation. Among those that have been validated, cytoskeletal genes, such as *actins*, and developmental genes, such as *Wnt *and *Hox *genes, show interesting expression profiles during regeneration.

**Conclusion:**

Our findings set the base for future studies into the molecular basis of intestinal regeneration. Moreover, it advances the use of echinoderms in regenerative biology, animals that because of their amazing properties and their key evolutionary position, might provide important clues to the genetic basis of regenerative processes.

## Background

All living organisms exhibit, to some extent, regenerative properties that allow them to deal with environmental events, physical trauma or diseases. Regenerative capacities have been studied in terms of stem cell recruitment, cell dedifferentiation, proliferation and migration, provision of specific regulatory/trophic factors, and expression or re-expression of the developmental program in adult animals [1]. However, the molecular/genetic basis of regeneration remains obscure.

Central to regeneration studies is the choice of the model organism, since metazoan species can show large variability in their regenerative capacities. In general, the ability to replace complex body parts decreases as one moves from the basal to the more highly derived taxa. However, even within the same phylum, not all animals are able to regenerate body parts, and not all tissues within a body can be equally repaired [2,3]. One problem in elucidating the molecular basis of regeneration has been that organisms with high regenerative capacities do not lend themselves easily to traditional experimental genetics. Only recently has progress been made to make possible the use of some of these model systems to dissect the genetic basis of regeneration [4]. Among these, invertebrate systems such as Hydra and planaria, and vertebrates, such as ascidians and amphibian urodeles, have gained particular attention. Nonetheless, regeneration research remains underpopulated, and there are whole phyla of organisms, showing very interesting regenerative behaviors where little molecular research has been performed [5]

Among deuterostomes, the regenerative potential is maximally expressed in echinoderms, animals that can quickly replace most injured organs. In particular, sea cucumbers (holothurians) are excellent models for studying organ regeneration since they can regenerate many of their organs and appendages. Our laboratory has been using an echinoderm, *Holothuria glaberrima *as a model organism for the last decade to dissect the process of how the digestive tract regenerates once it is eliminated by auto-evisceration. We have shown that following evisceration the new intestine regenerates from the free end of the remaining mesentery [6]. Initially, the mesenterial tip thickens forming a continuous rod-like structure that extends from the esophagus to the cloaca. During the second regeneration week, luminal epithelial cells from the esophagus and from the cloaca migrate into this tube, forming the mucosal layer and giving rise to the intestinal lumen. We have performed extensive studies at the cellular level showing the involvement of cell division, dedifferentiation, and migration in the regeneration of the intestine [6-11] as well as of events associated with the remodeling of the extracellular matrix [12].

We have also used the sea cucumber to explore the role of the genes that allow for such extraordinary regenerative processes [13]. Our approach has been to focus on target genes that have been associated with regenerative processes or identified in the regenerating tissues [14,15]. Similar gene by gene approaches have been used to study regeneration processes in other echinoderms, particularly in brittle stars and crinoids [16-20]. However, the regenerative capacities of the echinoderms have yet to be explored systematically using a large number of molecular tools [21]. In fact, one of the reasons that echinoderms have been sidelined in modern regeneration studies is the lack of genome-wide profiling approaches afforded by modern genomics [21]

We have now overcome this problem by determining the profile of gene activity during intestinal regeneration in *H. glaberrima *using microarray technology. For this, microarray slides were made using over 7000 ESTs that we previously identified from normal and regenerating intestine cDNA libraries [13]. Here we compare the gene expression profile of animals at 3, 7 and 14 days of regeneration, following the evisceration process typical of these organisms. Results show a large number of differentially expressed genes associated with intestinal regeneration. Some of these genes are homologues to metazoan genes associated with regenerative processes while many others might be novel sequences with little or no similarities to sequences in the databanks. To explore our results, we have focused on those sequences that are differentially expressed during early intestinal regeneration and that are associated with events previously shown to occur at the cellular level, namely developmental processes, cytoskeletal transformations and extracellular matrix remodeling. Our results make available the first overview of gene expression patterns in the regenerating intestine. More importantly, our results provide the basis for molecular studies aimed at exploring the molecular basis of regeneration in a group of animals that because of their remarkable properties and their key evolutionary position will provide important clues to the genetic basis of regenerative processes.

## Results

### Technical Analyses

Technical analysis showed that the microarrays performed as expected. First, the intensities of the 536 internal controls of the Agilent array perfectly matched the expected ones as shown in the regression model [see additional file 1]. Second, no significant variation was observed between raw data and normalized MA plots [Additional file 2], which means that there was no significant dye bias. Third, dye swap results showed extremely high similarity in their labeling pattern [additional file 3]. Since the dye swaps were made with different animal samples (biological dye-swaps), this implies that not only did dyes produced similar results, but that results from animals at the same regeneration stage were equivalent. Fourth, analyses of genes spotted in duplicate within the same array (technical replicates) showed almost identical fold changes, evidencing the strong reproducibility of our data.

### Gene expression profiles

#### A. Venn Diagrams

Microarray results showed dramatic differences in gene expression between regenerating and normal intestine and also among animals at different regeneration stages. Of the 14352 *H. glaberrima *probes in our microarray, 5915 showed similar expression in all groups, including normal (uneviscerated) and 3-, 7- and 14-days post-evisceration (dpe) regenerating intestines.

**Figure 1:**
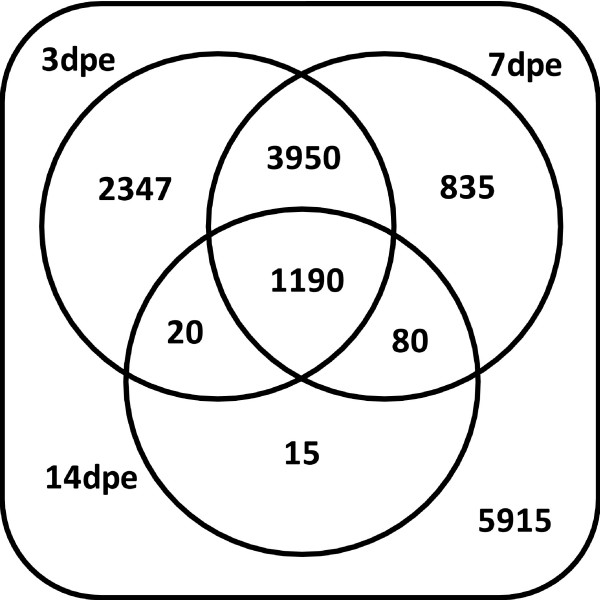
**Venn Diagram of the EST distribution**. 5915 probes do not show significant changes in expression when compared with normal tissues. The highest differential expression was found in both 3-dpe and 7-dpe stages (4439 differentially expressed spots); p < 0.01.

**Figure 2:**
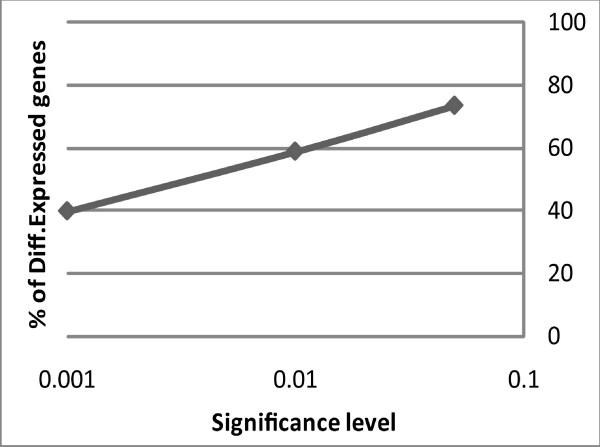
**Percentage of differentially expressed sequences at p < 0.001, 0.01 and 0.05**. At p < 0.001 only 39% of the sequences were significantly up or down regulated. *P*-values are shown in logarithmic scale.

Most of the differential expression was observed when normal and regenerating intestines were compared. A large number of probes, 8437 (aprox 58%) showed differential expression at a significance level of p < 0.01 (Figure 1). As expected, the number of differentially expressed spots at p < 0.05 was larger (73%) but even at p < 0.001 a significant number of genes (39%) still were found to be differentially expressed (Figure 2). Differential gene expression was also observed when comparisons were made among regenerating stages. The largest differences were observed in probes that changed specifically in the 3-dpe stage, whether they were differentially expressed only at 3-dpe (2347), at both 3- and 7-dpe (3950) or at 3-, 7- and 14-dpe (1190) stages compared to normal. A smaller number of probes was differentially expressed only at 7-dpe (835).

#### B. Volcano Plots

**Figure 3:**
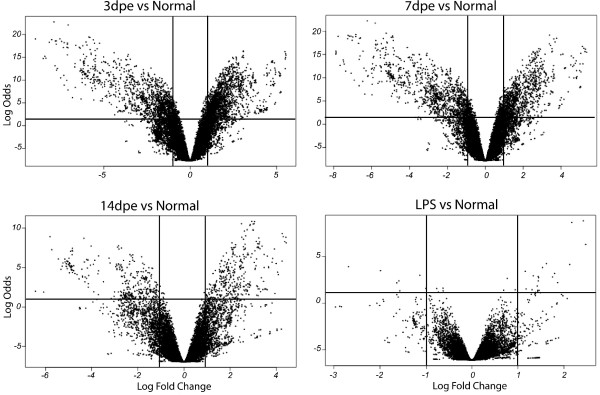
**Microarrays volcano plots**. Distribution of sequence expression between normal and normal injected with LPS, 3-, 7- and 14-dpe regenerating stages. Each plot shows the logarithm of the probability of the t-test as a function of the logarithm of fold change for each EST probe. Horizontal lines in each plot represent the nominal significant level of 0.001 for the *t*-student under the assumption that each gene has a unique variance. The vertical lines represent the limit of significance of the change in expression (fold change >2). Differentially expressed genes are located on the right (over-expressed) and left (under-expressed) top quadrants.

In order to have a graphical representation of the gene expression levels and their significance, we analyzed our results using volcano plots (Figure 3).

Results showed not only that a large number of genes are differentially expressed, but a high level of change and significance, mainly during the first week of regeneration. For example, at 3-dpe and 7-dpe the range of odds was significantly larger than at 14-dpe. Comparison with LPS showed a small number of genes being differentially expressed.

The top differentially expressed genes for 3-, 7- and 14-dpe stages of regeneration are shown on Tables 1, 2 and 3, respectively. Given that many probes have high significant *p*-values, we selected the top genes by two criteria: First, those probes with the highest *p*-value. Second, the fold change of the ratio of the intensities. The sequences were named either with their accession numbers (singlets) or with local identifications for multisequence contigs. All nucleotide sequences have been submitted to NCBI (taxonomy ID: 31192). Genes are shown in order of fold change and probability.

**Table 1:** Top 10 differentially expressed genes at 3dpe

**Top**	**Gene ID**	**Lib.**	BlastX*	**ESTs**	**Exp.**	**logFC**	**P.Value**
1	Contig 4863-1_			2	↑	5.541468	8.44E-11
2	ES726635	7d		s	↑	5.527128	6.06E-11
3	Contig 5300-1_			5	↑	4.879511	4.76E-10
4	Contig 3933-1_			2	↑	4.774727	3.03E-10
5	Contig 4702-1_			2	↑	3.091996	1.48E-10
6	Contig 798-1_			2	↑	3.080896	3.25E-11
7	ES725559	3d		s	↑	3.025192	1.2E-10
8	ES729465	N	TUBULIN A-1	s	↑	2.800781	5.86E-10
9	Contig 5344-1_		Cyclophilin	9	↑	2.797244	4.89E-10
10	ES725084	3d		s	↑	2.767621	5.84E-10
1	ES725883	7d		s	↓	-8.96457	1.67E-12
2	Contig 4571-1_			5	↓	-8.50941	1.83E-10
3	Contig 2794-1			2	↓	-7.88611	1.01E-14
4	ES728818	N		s	↓	-7.12955	1.32E-11
5	ES729355	N		s	↓	-6.66512	5.6E-10
6	ES729268	N		s	↓	-6.41638	1.32E-11
7	Contig 2489-1_			2	↓	-6.37315	1.7E-11
8	ES728532	N		s	↓	-6.18883	4E-10
9	Contig 5141-2_			6	↓	-6.17227	3.31E-10
10	Contig 5092-1_			2	↓	-6.00316	1.15E-10

Lib = cDNA library; FC = Fold Change; ↑: Up-regulated; ↓: Down-regulated; s:Singlet. *BlastX cut-off (1e-7)

**Table 2:** Top 10 differentially expressed genes at 7dpe.

**Top**	**Gene ID**	**Lib.**	**BlastX**	**ESTs**	**Exp.**	**logFC**	**P.Value**
1	Contig 4863-1_			2	↑	5.355039	8.63E-11
2	Contig 5300-1_			5	↑	5.04046	1.11E-10
3	Contig 2092-1_			5	↑	4.098951	1.29E-10
4	ES725788	3d		s	↑	3.153745	1.31E-10
5	Contig 3636-1_			7	↑	2.913074	4.39E-11
6	ES725084	3d		s	↑	2.796475	1.71E-10
7	Contig 5564-1_		TENASCIN R	2	↑	2.64799	1.39E-10
8	Contig 798-1_			2	↑	2.542767	8.18E-11
9	ES729465	N	TUBULIN A-1	s	↑	2.450837	1.31E-10
10	Contig 4696-1_		MURINOGLOBULIN	2	↑	2.308496	2.07E-10
1	Contig 4571-1_			5	↓	-7.89392	1.27E-10
2	ES725883	7d		s	↓	-7.76893	2.30E-12
3	ES729355	N		s	↓	-6.61787	1.96E-10
4	Contig 2794-1_			2	↓	-6.23549	3.57E-14
5	ES728532	N		s	↓	-5.89441	2.13E-10
6	ES728818	N		s	↓	-5.80657	3.42E-11
7	Contig 2489-1_			2	↓	-5.79542	1.44E-11
8	Contig 5092-1_			2	↓	-5.72744	5.99E-11
9	ES729268	N		s	↓	-5.69887	1.42E-11
10	Contig 5131-1_			2	↓	-5.44947	3.85E-11

Lib = cDNA library; FC = Fold Change; ↑: Up-regulated; ↓: Down-regulated; s: Singlet.

**Table 3:** Top 10 differentially expressed genes at 14dpe.

**Top**	**Gene ID**	**Lib.**	**BlastX**	**ESTs**	**Exp.**	**logFC**	**P.Value**
1	Contig 4863-1_			2	↑	4.432583	1.34E-07
2	Contig 5300-1_			5	↑	3.927607	3.05E-07
3	Contig 3636-1_			7	↑	3.386883	2.09E-07
4	Contig 5164-1_			5	↑	3.144439	1.11E-06
5	Contig 3791-1_		NM23 (NDK)	3	↑	2.953193	5.68E-07
6	Contig 4216-1_			2	↑	2.769543	5.21E-07
7	Contig 3895-1_			3	↑	2.735413	2.20E-08
8	ES727517	7d		s	↑	2.606467	8.29E-07
9	ES725788	3d		s	↑	2.576167	2.27E-07
10	ES726378	7d	LAMININ, A-4	s	↑	2.415549	7.96E-07
1	ES728532	N		s	↓	-5.80681	5.86E-08
2	Contig 5141-2_			6	↓	-5.16706	1.13E-06
3	ES725817	7d		s	↓	-4.33374	7.32E-08
4	ES725924	7d		s	↓	-4.21937	1.20E-06
5	ES725893	7d		s	↓	-4.15444	1.27E-06
6	ES726019	7d		s	↓	-4.02758	2.07E-07
7	ES729325	N		s	↓	-3.70124	7.48E-07
8	ES727944	7d	LEU-RICH REP.K-2	s	↓	-2.87982	2.40E-07
9	Contig 2398-1_			s	↓	-2.82164	3.09E-07
10	Contig 76-1_		SOMA FERRITIN	76	↓	-2.34468	6.22E-07

Lib = cDNA library; FC = Fold Change; ↑: Up-regulated; ↓: Down-regulated; s:Singlet.

Among the genes in this top 10 list that show known identities, were *cyclophilin, laminin, alpha tubulin, tenascin-R, ferritin. murinoglobulin *and *NM23*. Interestingly, most of the differentially expressed genes showed no significant similarity (e-value < 1.00E-07) to other sequences in publicly available nucleotide or protein databases. Unknown genes, such as Contig 4863-1 and 5300-1, were up-regulated at 3-, 7- and 14-dpe, while the singlet ES728532 showed down-regulation at all regeneration stages. On the other hand, some genes were specific for one stage such as Contig 3933-1 and Contig 4702-1 at 3-dpe. However, these genes were significantly differentially expressed but with higher p-values at other time points.

An alternate strategy to focus on gene groups that are differentially expressed at particular regeneration stages is to compare gene expression among regeneration stages (3-, 7- and 14dpe) and not directly to the normal intestine. To do this, we used the expression profile at 7-dpe as reference (up-regulated genes at 7dpe are considered as those whose expression is down-regulated at normal, 3 and 14dpe- stages; contrary, down-regulated genes are those whose expression is up-regulated at normal, 3 and 14 dpe). Seventeen genes (corresponding to 26 ESTs represented by 55 probes in the microarray) were found to be up-regulated at 3-dpe when compared with 7dpe (Table 4). The number of genes with up-regulated expression at 7- and 14-dpe stages was 24 and 13, respectively (Table 5 and 6).

**Table 4:** Upregulated genes specific to 3dpe

**Gene ID**	**BlastX**	**p.value**
Contig 739 2_		9.00E-05
Contig 66 1_		0.00016
Contig 5453-1_		0.00021
Contig 739 1_		0.00031
ES727789		0.00316
Contig 4797-1_		0.00334
Contig 4797-1_		0.00438
Contig 3640-1_		0.0058
Contig 798 1_		0.00669
Contig 3158-1_		0.00705
Contig 3640-1_		0.00784
Contig 3171-5_		0.00948
ES725288		0.01487
Contig 5652-1_	60S RIBOSOMAL L27	0.0157
ES727261		0.02239
Contig 5537-1_		0.02847
Contig 2300-1_		0.04107

**Table 5:** Upregulated genes specific to 7dpe

**Gene ID**	**BlastX**	**p. alue**
Contig 3895-1_		9.00E-05
ES729486		1.00E-04
ES727178		0.00016
Contig 771-1_	60S RIBOSOMAL PROTEIN L5	0.00032
ES726371		0.00044
Contig 5306-1_	APOLIPOPHORINS PREC.	6.00E-04
Contig 4810-1_		0.00062
ES727405		0.00065
Contig 3525-1_	EPENDYMIN REL. PROT.2	0.00073
Contig 89-1_6981		0.00169
ES727912	BONE MORPHOGENETIC PROT.1	0.00204
Contig 89-1_		0.00204
AY383544	EPENDYMIN REL. PROT. 1	0.00397
ES726186		0.00508
ES714731		0.00646
ES725228		0.00721
Contig 5342-1_		0.00906
ES728886		0.01851
ES729552	FAT (Cadherin)	0.01851
ES726575		0.02755
ES716626		0.02822
ES881026		0.03701
ES727189		0.04123
Contig 2092-1_		0.04342

**Table 6:** Upregulated genes specific to 14dpe

**Gene ID**	**BlastX**	**p.value**
Contig 2794-1_		6.00E-05
ES728818		0.00166
Contig 3191-1_		0.00176
ES725883		0.00551
ES729268		0.01215
ES728850		0.03556
Contig 4571-1_		0.04393
ES729033		0.0094
ES727539		0.02361
ES728538		0.00371
ES729914		0.0106
ES725898		0.03392
ES726811		0.02018

#### C. Validation of Microarray Results by RT-PCR

Gene-specific relative RT-PCRs were performed to validate the expression profiles of fifteen genes identified in the microarray as differentially expressed. The sequences of the primers used are shown on Table S1. Validation included genes with putative identity (*Wnt-9, Tensc-R, MMP-15, MMP-11, MMP-14, Actin 1, Actin 2, Actin 3 *and *Hox12*). Most of these genes have been previously associated with wound-healing, development and regeneration in other animal species. The second group consisted of genes without identity (putative novel genes-Unknowns E, F and G, Contig 4874-1, Contig 5242-1 and Contig 5501-1). The validated genes include over-regulated and down-regulated sequences of the microarray.

**Figure 4:**
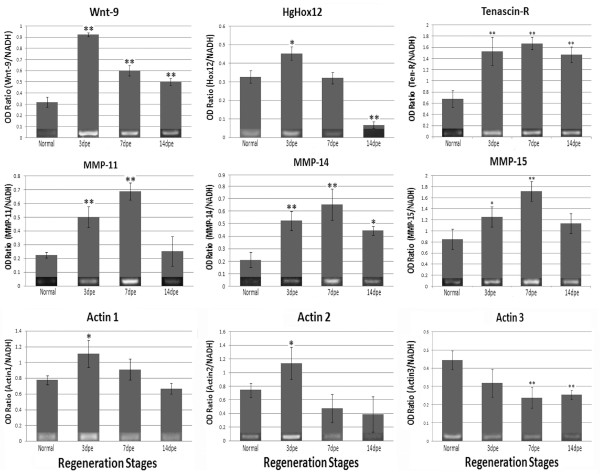
**RT-PCR profiles of *Wnt-9, Hox12, Tenascin-R, MMP-11, MMP-14, MMP-15, Actin 1, Actin 2 *and *Actin 3 *in normal and regenerating intestines**. The values were normalized against NADH. This set of genes showed up-regulation in the first week of regeneration except for *Actin 3 *that showed down-regulation during regeneration. Interestingly, all extracellular matrix (ECM) related genes showed the peak of expression at 7-dpe. Moreover, *Wnt-9*, a developmental gene, was up-regulated during all stages. Values represent the mean ± S.E.M. of 7 biological samples (individual animals) per stage. Paired t-test for mean comparison was used. Significance levels were *:p < 0.05, and **:p < 0.01. Images at the base of the bar are RT-PCR pictures.

**Figure 5:**
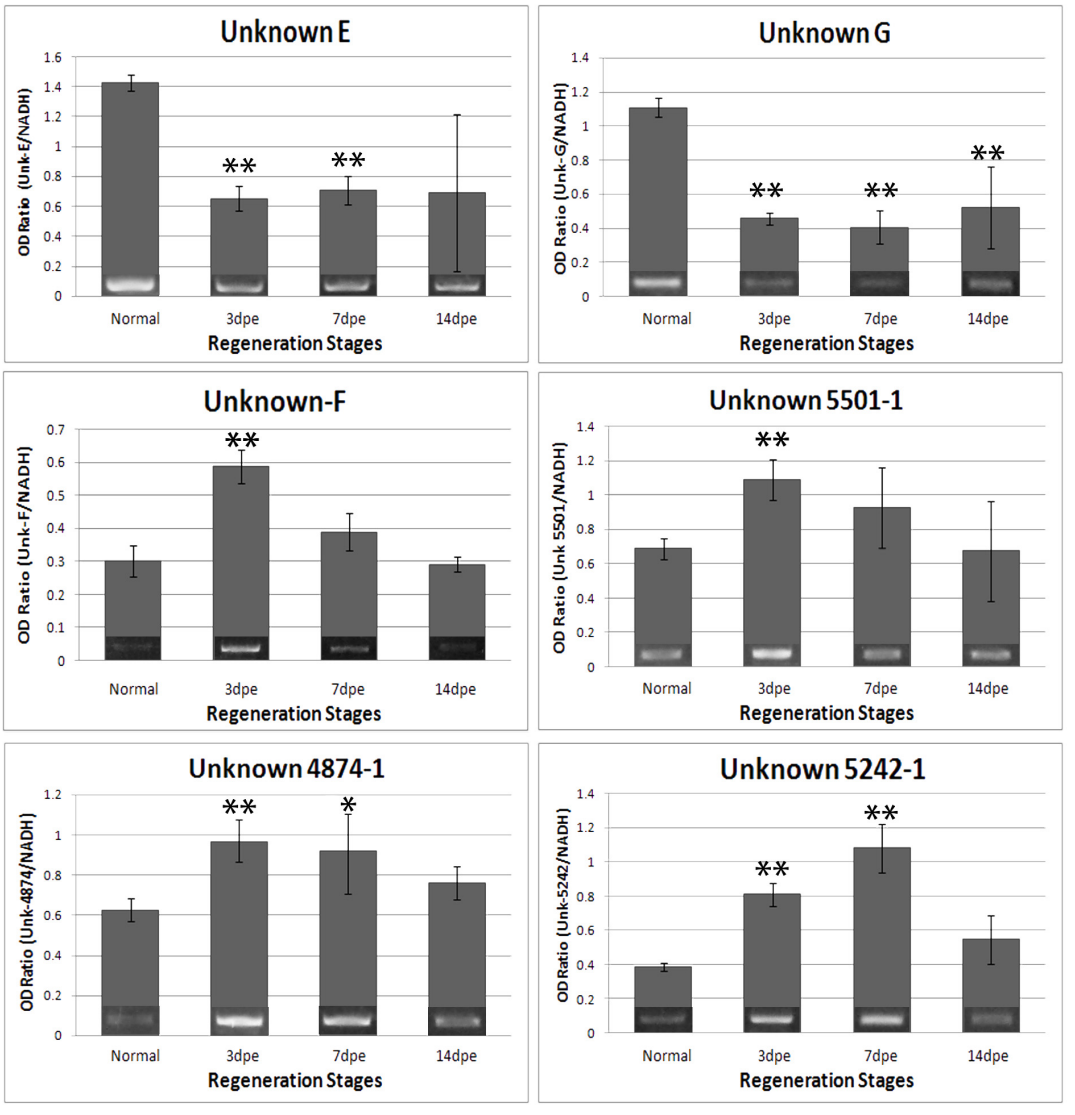
**RT-PCR profiles of 6 novel gene sequences in normal and regenerating intestines**. The values were normalized against NADH. This set of genes was mainly differentially expressed in the first week of regeneration, except by unknown-G that keeps down-regulated at 14dpe. Three of the four up-regulated genes showed the peak of expression at 3dpe and decrease the expression until 14dpe. Only unknown 5242-1 showed the peak at 7dpe but even at 3dpe the up-regulation was highly significant when compared with normal tissues. Values represent the mean ± S.E.M. of 7 samples per stage. Paired t-test for mean comparison was used. Significance levels were: *:p < 0.05, and **: p < 0.01. Images in the base of the bar are RT-PCR pictures

The expression profiles of the 15 genes that were validated using RT-PCR confirmed the robustness of the microarray results (Figure 4 and 5). Eleven out of the 15 genes showed exact correlation in their expression profiles between RT-PCR and microarrays and very similar significance values in the statistical analyses. For example, unknown F showed up-regulation at 3-dpe and decreased gradually at 7- and 14-dpe. The unknowns 5501, 4874 and 5242 showed an exact match between the microarray and the PCR results. On the other hand, the correlation was not perfect for four genes (*actin 1, 2 *and *3*, and *Hox 12*). These genes showed some correlations at particular stages, such as observed for *actin 1 *and *2 *and *Hox 12 *at 3-dpe. At other stages some of the RT-PCR results although not significant do showed a trend that resembled the microarray results (see *actin 3 *at 3-dpe). Overall the correlation of RT-PCR and microarray results was 84%, with only 7 of the 45 validated time points not showing the same profile between the microarrays and the RT-PCR. A direct comparison of the RT-PCR and the microarray results is shown on Table 7.

**Table 7:** Significance obtained from microarray and PCR at 3, 7 and 14dpe

**Gene ID**	**3dpe Microarray**	**RT-PCR**	**7dpe Microarray**	**RT-PCR**	**14dpe Microarray**	**RT-PCR**
Wnt-9	↑ 7.00E-05	6.00E-05	↑ 4.00E-05	4.00E-03	↑ 0.013	0.009
Tensc-R	↑ 1.00E-05	5.00E-03	↑ <1.00E-05	4.00E-03	↑ 1.00E-4	8.00E-03
MMP-11	↑ 4.00E-05	5.00E-03	↑ <1.00E-05	6.00E-05	0.15	0.536
MMP-14	↑ 2.00E-03	1.00E-03	↑ 2.00E-04	2.00E-04	0.14	0.02
MMP-15	↑ 0.0485	0.0475	↑ 3.00E-03	4.00E-03	0.23	0.09
Unk- E	↓ 3.00E-05	4.00E-05	↓ 7.00E-04	1.00E-04	0.45	0.09
Unk- F	↑ 5.00E-04	3.00E-03	0.12	0.06	0.85	0.84
Unk- G	↓ <1.00E-05	5.00E-04	↓ <1.00E-05	2.00E-04	↑ 2.00E-4	4.00E-03
Unk- 4874-1	↑ 3.00E-05	1.00E-03	↑ 0.0011	0.02	0.1689	0.196
Unk- 5242-1	↑ 1.00E-04	4.00E-05	↑ 4.00E-04	1.00E-05	0.19	0.06
Unk- 5501-1	↑ 7.00E-05	6.00E-03	0.123	0.138	0.36	0.933
Actin-1	↑ <1.00E-05	0.04	↑ <1.00E-05	0.281	↑ 2.00E-4	0.276
Actin-2	↑ 4.00E-04	0.034	↑ 4.00E-04	0.116	0.05	0.09
Actin-3	↓ 1.00E-04	0.26	↓ 1.00E-04	0.005	↓ 0.013	0.005
Hox-12	↑ 2.00E-03	0.033	↑ 0.035	0.48	0.54	↓ 0.0016

↑: Up-regulated; ↓: Down-Regulated

#### D. Cluster Analysis

**Figure 6:**
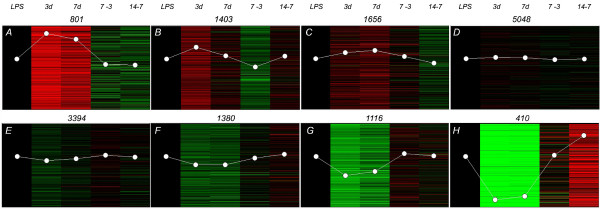
**Cluster analysis of the microarray expression profiles for 15208 probes (All minus 536 controls)**. The 5 columns show the following comparisons: LPS (Red: "R") vs. Normal (Green: "G"): 3dpe(R) vs. Normal(G); 7dpe(R) vs. Normal(G); 7dpe(R) vs. 3dpe(G); 14dpe(R) vs. 7dpe(G). The number of genes in each cluster is shown above the bars. Since arrays were normalized against the LPS vs Normal results, the first column appears empty. Colors represent the level of expression of the gene, red for up-regulated and green for down-regulated; White spots represent the average of all the genes per cluster with "y" axis as a relative measure of expression levels.

Cluster analysis was performed on the microarray results using hierarchical (not shown) and non-hierarchical methods in order to reveal several distinct profiles of expression (Figure 6).

Cluster # A contains probes that were highly over-expressed at 3- and 7-dpe when compared to normal intestine, and whose expression levels are slightly higher in 3- than in 7-dpe and are further decreased at 14-dpe.

Cluster # B contains probes that were moderately over-expressed at 3-dpe when compared to normal and whose expression levels decreased at 7-dpe. The expression levels of these probes at 14-dpe were similar to those at 7-dpe.

Cluster # C contains probes that were over-expressed at 3-dpe but whose expression peaked at 7-dpe, followed by a decrease in expression levels at 14-dpe.

Cluster # D contains probes that showed no significant differences in expression at any stage when compared to normal intestine.

Cluster # E contains probes that were slightly under-expressed at 3-dpe when compared to normal. The expression level of these probes was higher at 7- than at 3-dpe and somewhat similar between 7- and 14-dpe.

Cluster # F contains probes that were slightly under-expressed at 3- and 7-dpe when compared to normal and whose expression levels increase at 14-dpe.

Cluster # G contains probes that were moderately under-expressed at 3-dpe when compared to normal and whose expression levels slightly increase at 7- and 14-dpe.

Cluster # H contains probes that were highly under-expressed at 3- and 7-dpe and whose expression levels showed a moderate increase at 14-dpe.

#### E. Functional category analysis

**Figure 7:**
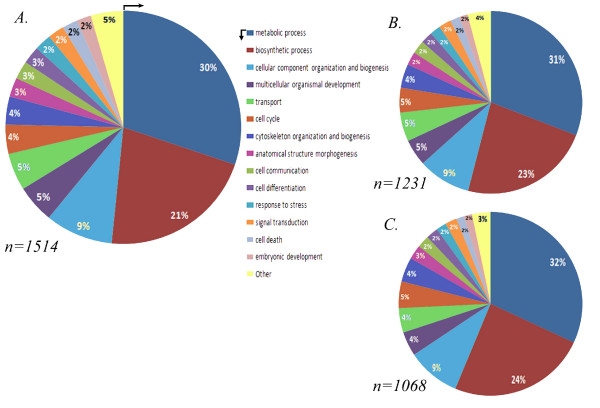
**Distribution of gene function (GO) for ESTs analyzed in the microarrays**. GO values of all ESTs printed on the microarray (A). Percentage of ESTs differentially expressed at 3-dpe (B) or 7-dpe (C). When compared with all the GOs, the 3- and 7-dpe distribution GOs showed no significant differences other than a small decrease in the number of GO results. The number of GOs results for each particular stage is shown in the bottom-left corner. The color code starts at 12 o'clock in the list order.

We submitted all sea cucumber sequences to BlastX and parsed the results using a threshold of significance e-value < 1.00E-05. Approximately 58.1% of the sequences (3651) were "unknowns" that showed no similarity to subjects in the SwissProt database. On the other hand, 41.9% of the sequences (2636) had similarity with database sequences. Of these, 169 showed similarity with hypothetical proteins, most of them from the sea urchin *S. purpuratus*. GO searches  were performed with all the blast results and 1514 unique sequences were found with at least one reported ontology (Figure 7A).

In order to determine if particular gene functions could be associated exclusively with specific regeneration stages, diagrams were done with only those genes that showed differential expression between 3-, 7- or 14-dpe and normal. At 3-dpe the GO search showed 1231 results while 1068 were found for 7-dpe (Figure 7B and 7C). The distribution of the GO was highly similar between all sequences and regeneration stages. For example, in all cases sequences associated with metabolic and biosynthetic processes corresponded to half of the GO results.

#### F. Individual gene expression

In addition to the global view of gene expression provided by microarray analyses, individual gene expression patterns can be determined. We have focused on various genes that have been associated with regenerative processes. For this we only chose those genes whose similarity to genes from other animals was sufficiently high (e-value < 1.00E-07) to be confident that they correspond to holothurian homologues. These genes are classified and presented in three main groups in relation with their function: a) Developmental genes, b) Cytoskeletal-related genes and c) Extracellular matrix-related genes.

##### a). Developmental genes

Many developmental gene pathways have been found to occur or be activated during regenerative processes [22,23]. Thus, we have focused on the expression profile of development-associated genes to explore their roles during intestinal regeneration. Table 8 shows the expression profile of well-known developmental genes in the microarray.

**Table 8:** Expression profile of developmental genes in the sea cucumber *H. glaberrima *during regeneration.

**Gene**	**3-dpe**	**7-dpe**	**14-dpe**
Hox 1	-	-	-
Hox 2	-	-	-
Hox 3	-	-	-
Hox5	-	-	↑*
Hox 9	↑*	↑**	-
Hox10	↑**	↑**	-
Hox 11	-	-	-
Hox 12	↑**	↑*	-
Hox 13	-	-	-
TGFB-inducible LNR42	↑**	-	-
Krueppel-like	↓*	-	-
Wnt-9	↑**	↑**	↑*
BMP-1	-	↑**	-
Forkhead box prot. K1	↓*	-	-
Myotrophin	↓**	↓**	-

↑: up-regulated; ↓: down-regulated; *:p < 0.05; **:p < 0.01

In general, differential expression of these genes mainly occurred at early stages of regeneration. Most of the genes are activated or inhibited at 3-dpe (8 genes, being 5 of them highly significant) and 7-dpe (6 genes, being 5 highly significant). In contrast, at 14-dpe only two developmental genes were differentially expressed, both just at *p *< 0.05. Hox genes stand out among the group of development-associated genes, and our group has previously identified them in the holothurians [24]. Four of these genes appeared to be over-expressed, although their expression profile differed. *Hox 9, 10 *and *12 *were over-expressed at 3- and 7-dpe and returned to normal levels at 14-dpe. However, while *Hox 10 *displayed the same levels of over-expression at 3- and 7-dpe, *Hox 12 *and *9 *showed peaks of expression at 3-dpe and 7-dpe, respectively. *Hox 5 *showed a different pattern of expression altogether being over-expressed only at 14-dpe. Other development-associated genes were also found to be differentially expressed in our microarrays during intestinal regeneration. Once again the expression profile differed among them. For example, *Wnt 14 *was found to be over-expressed at 3-, 7- and 14-dpe, while *BMP-1 *was only up-regulated at 7-dpe. In contrast, *forkhead box K1 *was down regulated at 3-dpe and, similarly, *myotrophin *was down-regulated at 3- and 7-dpe. Finally, not all development-associated genes showed changes in expression. For example, *Kruppel-like factor 2 *remained at similar levels of expression during the first two weeks of regeneration.

To validate the developmental gene results we chose to study the expression of *Hox 12 *and *Wnt 9 *by RT-PCR. The sequences for these genes were obtained from the 7-dpe library and the complete sequence of *Hox 12 *has been previously characterized by our laboratory [25] RT-PCR showed that genes were over expressed during early regeneration and their levels decreased during the second week of regeneration.

##### b). Cytoskeletal Proteins

Regeneration induces cellular changes that have been previously reported by our group. Among these are processes of dedifferentiation and migration that are associated with the formation of the new intestinal primordium during early regeneration [7-9]. Such processes are associated with changes in the expression of cytoskeletal proteins. Thus, it is expected that these changes would be reflected in the expression of mRNAs coding for cytoskeletal proteins. We have focused on the expression of seven cytoskeletal genes found within our EST database (Table 9). These include three actin family members, four tubulin family members, two myosin genes and one gelsolin gene. Although actin and tubulin isoforms are highly similar at the nucleotide level of the open reading frame (ORF), we identified ours based on differences in their 3'-unstranslated (UTR) region. The expression profile of these cytoskeletal mRNAs differed widely. Some were over-expressed during the first 2 weeks of regeneration (*Actin 1, Actin 2, Tubulin alfa and alfa-1*), others were under-expressed (*Actin 3, Myosin-11 *and *gelsolin precursor*) while others showed no significant changes in their expression (*tubulin-beta*). Of particular interest were those sequences that showed a specific change at 3-dpe and appeared to only change slightly or not at all at other regeneration stages (*tubulin 3 *and *myosin light 3F chain*), since these are more likely to play a role in specific processes occurring during intestinal regeneration.

**Table 9:** Expression profile of cytoskeletal genes in the sea cucumber *H. glaberrima *during regeneration.

**Gene**	**3-dpe**	**7-dpe**	**14-dpe**
Beta-Tubilin	-	-	-
Alfa-Tubulin 1	↑**	↑**	↑**
Alfa-Tubulin 2	↑**	↑**	↑**
Alfa-Tubulin 3	↑**	↑**	-
Myosin-11	↓**	↓**	↓*
Myosin-Light 3F	-	↑*	-
Actin-1	↑**	↑**	↑**
Actin-2	↑**	↑**	↑*
Actin-3	↓**	↓**	↓*
Gelsolin-precurs	↓**	↓**	↓**

↑: up-regulated; ↓: down-regulated; *:p < 0.05; **:p < 0.01

Some of these sequences were validated by RT-PCR. In particular, the differential expression profiles of *actins 1 *and *2 *that are over-expressed during early regeneration stages, while *actin 3 *is down-regulated, was clearly documented in our RT-PCRs.

##### c). ECM genes

We have previously described dramatic remodeling of the ECM and the involvement of MMPs during intestinal regeneration [12]. In this respect we searched for sequences in our database that might be associated with the ECM changes. Seven such genes were found, four of them corresponding to ECM molecules (*Echinonectin, Collagen alfa-1, Laminin alpha *and *Tenascin-R*) and three corresponding to matrix metalloproteases (MMPs) (15, 11, and 14) (Table 10). In this group, all analyzed genes showed up-regulation when compared with normal tissues. The mRNAs for the four ECM molecules showed over-expression during the first two regeneration weeks. Different to ECM molecules, MMPs were over-expressed at 3- and 7-dpe but not at 14-dpe, with *MMP 15 *showing low significance at 3- but high at 7-dpe. The four genes from this group that were validated (Figure 5) showed strong correlation between RT-PCR and microarray results.

**Table 10:** Expression profile of ECM-related genes in the sea cucumber *H. glaberrima *during regeneration.

**Gene**	**3-dpe**	**7-dpe**	**14-dpe**
Echinonectin	↑**	↑**	↑**
Collagen alfa-1(V)	↑**	↑**	↑**
tenascin-R	↑**	↑**	↑**
Laminin alfa 4 precurs	↑**	↑**	↑**
Laminin alpha 1	↑**	↑**	↑*
MMP 15	↑*	↑**	-
stromelysin-3 (MMP 11)	↑**	↑**	-
MMP-14	↑**	↑**	-

↑: up-regulated; ↓: down-regulated; *:p < 0.05; **: p < 0.01

##### d). Novel sequences

As mentioned earlier many of the sequences in our microarray slides were unknowns. That is, sequences that showed no similarities to those in databanks. In many cases this was due to the sequence being either too short or being part of the mRNA's UTR. However, in some cases the sequence was long enough to contain a significant ORF that, in principle, should have shown similarities to other proteins when submitted to BLAST searches. These are the most interesting sequences to our research group, particularly those that were differentially expressed during regeneration, since they might correspond to novel holothurian-specific sequences associated with their striking regenerative capacities.

One strategy that we explored to study these unknowns was to focus on contigs. That is, those ESTs that shared overlapping sequence identity among them, and that together formed a composed sequence. The number of ESTs from a specific library (stage of regeneration) found to form a contig can be correlated with the expression level for that sequence at the stage from where the cDNA library was made. Therefore, an indirect measurement of the level of gene expression can be inferred from the number of identified ESTs from each library that formed contigs. We identified 596 multi-sequence contigs in our EST databank that had at least two assembled ESTs. Of these, 85.4% had ORFs larger than 100 nt (~33aa), and 46.5% had ORFs larger than 195 nt (~65aa). Only 27 contigs had no ORFs made up of at least 30 nt. We focused on contigs that showed over-expression at 3- and 7-dpe that were composed by at least three different ESTs and where all the EST probes showed similar results in the microarray. Sixteen of these unknown contigs are shown on Table 11. Their putative ORFs ranged from 98 to 794 nucleotides in length and they were represented by three to twenty-nine different ESTs. As expected, down-regulated contigs at 3-dpe appeared in the H and E clusters, while the up-regulated contigs at 3- and 7-dpe were located mostly in the A and B clusters. Moreover, some of these genes were validated with RT-PCR (Figure 6) showing high consistency in their expression profiles (Table 3).

**Table 11:** Novel genes differentially expressed during regeneration in H. glaberrima.

**ESTAP (cluster-assembly)**	**Val.**	**# Probes (ESTs)**	**3d D.E.**	**Orf Size (nt)**	**Orf-Start**	**Orf-end**	**G.O. Cluster**
Contig 2748-1_Unk I		13 (5)	↓	371	719	1090	E
Contig 2794-1 _		5 (2)	↓	185	364	546	H
Contig 4277-8_Unk E	√	50 (26)	↓	110	1	111	E
Contig 4910-1_Unk H		9 (4)	↓	98	89	187	E
Contig 5141-2_Unk G	√	34 (13)	↓	209	144	353	H
Contig 293-1_		8 (3)	↑	737	46	783	D
Contig 4216-1_		7 (3)	↑	143	70	213	C
Contig 4766-1_Unk C	X	36 (18)	↑	365	91	456	A
Contig 4791-1_Unk F	√	35 (18)	↑	269	69	338	B
Contig 4860-1_Unk D	X	16 (8)	↑	242	48	290	B
Contig 4874-1_	√	16 (7)	↑	317	89	406	A
Contig 4911-1_Unk B		58 (29)	↑	218	1	219	A
Contig 5242-1_	√	9 (3)	↑	794	61	855	B
Contig 5501-1_	√	10 (4)	↑	125	1	126	B
Contig 5701-2_		6 (3)	↑	230	119	349	B

(√: validated genes in this paper; x: validated previously [13]

##### e). Other sequences

Our microarray chip included the sequences of 75 ESTs from another sea cucumber *A. japonicus*, and 329 sequences from genes of the sea urchin *S. purpuratus*. Only five of the sea cucumber (*Collagen Alpha-1 (CA1); Cytoskeleton actin (cAct); Fibrinogen B precursor (Fib-Bp), Heat Shock protein 90b (HSP-90) and Senescence associated protein (SAP)) *showed significant differences in expression in regenerating animals. Two of *A. japonicus *differentially expressed ESTs were underexpressed during regeneration (*Fib-Bp *and *SAP*), while the remaining three were overexpressed. Interestingly, four were similar to the *H. glaberrima *ESTs and followed the same expression profile (*Fib-Bp *was the different one). In all cases the probes of these other organisms showed similarity with the C-terminal region of the *H. glaberrima *sequences. The only sequence from the sea urchin that changed during regeneration was a hypothetical protein (Glean3-07946) showing a decrease in its expression at 3- and 7-dpe. The 24 sequences from zebra fish, human, rat, mice frog and axolotl behaved as expected; none showed any significant labeling or differential expression.

## Discussion

We have now shown by using microarray technology that a large number of genes, represented by EST sequences, are differentially expressed during sea cucumber intestinal regeneration. Nonetheless, microarray results are not fool-proof and must be validated by other methods. An indirect way of validating our results is to compare this microarray data to previous work from our laboratory showing individual gene expression patterns. Two examples of these are the expression of serum amyloid A (SAA) [14] and of ependymin [15], both of which have been well studied in the laboratory using a range of molecular techniques. SAA was shown by Northern blot analysis to be over-expressed during regeneration, with a peak during the second week[14]. Our microarray results also show SAA over-expression during intestinal regeneration, although the expression peak somewhat differs, occurring during the first week. This difference might be due to the fact that SAA is also associated with the immune status of the animals [26] therefore this status might differ between the animals used in the present experiments from those used for the Northern experiments performed years ago. On the other hand, the expression of ependymin, a gene associated with regeneration and plasticity, was previously shown to increase during intestinal regeneration [15]. These results were obtained with the highly accurate technique of quantitative Real Time PCR, showing a peak in expression at 7-dpe and no change at 3- or 14-dpe. These results are closely reproduced in our microarray analyses which show significant differences between 7-dpe animals and uneviscerated, but no differences at 3- or 14-dpe.

The comparison of RT-PCR results and microarray data obtained for fifteen selected ESTs provides a direct corroboration of their differential expression during intestinal regeneration. These results show that 11/15 of the sequences behave essentially the same whether measured by microarray or RT-PCR. Moreover, even the statistical significance of the result is within the same ballpark figure. The differences between RT-PCR and microarray results of the four remaining sequences (*Hox 12, actins 1, 2 *and *3*), is mainly due to the RT-PCR results not being statistically significant, although in some cases they follow the same trend. It is interesting that these sequences are among the ones known to have closely related sequences in the genome (multiple actin genes and Hox genes), thus suggesting that the lack of correlation at some stages might be due to primers hybridizing with other sequences and increasing the background noise, making the RT-PCR results less sensitive than the microarray.

### • Why do so many genes show differential expression?

One of the most striking results from our microarray experiments, is that a high percentage of the spotted ESTs were found to be differentially expressed. Even when considering a very high level of significance (p < 0.001) more than a third of the ESTs showed differential expression. This contrasts with other regeneration studies where a smaller number of genes were found. For example, in studies of zebrafish heart regeneration, only about 5% of the assayed genes were found to be differentially regulated [27] and between 8 and 14% in studies of liver regeneration in rodents [28,29].

The reasons for these differences might be due to two factors. First, most of our ESTs were obtained from cDNA libraries of regenerating intestine, thus the original pool of ESTs is probably biased toward those genes that are highly expressed in regenerating tissues. This was confirmed by the results themselves: the set of probes differentially expressed at 3dpe and 7dpe were 81.25% from the 3- and 7-dpe libraries and only 18.75% from the Normal library. In contrast, 42% of the down-regulated sequences come from the Normal library. Second, the process of intestinal regeneration that we are studying is complex and involves organogenesis, not merely wound healing. Furthermore, the intestine is an organ with multiple cell types and embryological origins. The intestine has components of the three embryonic germ layers, the enteric nervous system being an ectodermal derivative, the luminal epithelium an endodermal derivative and the muscular layer and submucosa being mesodermal derivatives. Therefore, when compared to a process such as heart or liver regeneration, where fewer cell types or more limited regeneration is involved, a larger number of genes might be necessary to be modulated to achieve the formation of the new intestine. Moreover, in the study of zebra fish heart, regeneration was limited to the ventricle and only to a portion of the organ since only 20% of the ventricle was removed. This contrasts largely from our studies in the sea cucumber where the new intestine must be completely formed from the remaining mesentery. In fact, a large number of differentially expressed genes was also found in another organism with high regenerative potential, the salamander *Ambyostoma mexicanum*. In this species when the tail, including the spinal cord segment is amputated, 76% of the genes in the microarray were differentially expressed [30]. It is interesting that they also found that even when increasing the stringency of the statistical analyses the number of differentially expressed genes (35%) was still significant. Thus, regeneration in deuterostomes imply the upregulation and downregulation of a large number of genes, making the search for "master regeneration genes", much more difficult than anticipated.

In view of this difficulty, we have developed certain strategies aimed at singling out those genes that might play important or crucial roles in regeneration. First, is to focus on genes that are differentially expressed at the 3-dpe stage. Previous experiments from our laboratory have shown that the cellular processes associated with intestinal regeneration occur early in intestinal regeneration. By 7-dpe, the blastema-like structure that will be the primordium for the new intestine is already formed [6]. Thus, genes associated with initiating regeneration must be regulated earlier than at 7-dpe. In this respect, some of the most likely candidates will be those genes that are differentially expressed between the 3- and 7-dpe stages. Second, focus on those genes that are most highly differentially expressed, such as those that were identified on our Top ten analyses. Third, look at sequences that form contigs. Although the presence of multiple contigs might be explained by several factors (some of them technique-related), they could be reflecting the abundance of gene expression in the original cDNA libraries, and therefore at the stage from where the library was made. Fourth, is to aim at those genes that have been previously associated with organogenesis in other species (see the following section). Finally, we expect that novel genes associated with regeneration will be characterized from our data. Thus, those sequences, and in particular those contigs that show ORFs with no similarity to sequences in databases are the strongest candidates to be pursued.

### • How do microarray results correlate with organogenesis processes that occur during embryonic development?

#### Developmental Genes

The finding of genes associated with embryonic development being differentially expressed during regeneration is not surprising since developmental gene pathways have been found to occur or be activated during regenerative processes [22,23]. Thus, these genes and signaling pathways provide excellent targets to study the molecular events of intestinal regeneration.

One of the most studied groups of genes is the Hox genes, which encode transcription factors that specify the body axis during embryonic development. Hox genes have been implicated in various regeneration processes, including the regeneration of amphibian tails and limbs [31-33], hydra [34], rat liver [35], and zebrafish tail [36] among others. This expression can be spatially or temporally specific in regenerating tissues [37,38]. It is particularly interesting that *Hox12 *had previously been found in our regenerating intestine cDNA library and that there is some evidence for homeobox gene involvement in echinoderm regeneration [20]. The finding that the most posterior genes (*Hox 9*, *10 *and *12*) are the ones that showed an increase in expression during intestinal regeneration coincides with findings in the mammalian digestive tract where Hox genes are known to be expressed in a rostral-caudal gradient, and the Hox genes near the 5' end are expressed in the posterior digestive tract, i.e. intestine [39]. These are the same genes that when mutated cause defects in the development of the posterior digestive tract [40,41].

Similarly, the finding that a Wnt homologue (*Wnt-9*) was overexpressed in the regenerating intestine during the first two weeks of regeneration coincides with published data on this molecular family. Wnt molecules are extracellular molecules that bind to their receptors and activate a signaling pathway that includes other known proteins, such as B-catenin. In recent years, Wnt pathways have been increasingly associated with regenerative phenomena. Wnt was found to be involved in blastema formation of the regenerating limbs of anuran tadpoles [42] and in lens regeneration in newts [43]. In mammals, Wnt has been studied in bone [44], hair follicle [45] and deer antler regeneration [46] among others. Liver regeneration was retarded in the absence of beta-catenin [47]. Wnt apparently plays a key role in the control of intestinal stem cell proliferation and differentiation [48]. Thus, it will be of great interest when the holothurian *Wnt-9 *expression is localized to determine which intestinal cell types are expressing this protein. Wnt pathways have also been involved in other invertebrate regeneration models. In planaria, Wnt is necessary for proper brain pattern formation [49] and B-catenin for antero-posterior axis formation during regeneration [50].

Lastly, the family of bone morphogenetic proteins (BMPs) has been increasingly associated with regenerative phenomena. In newts, BMPs have been associated with the potential of the ventral iris to regenerate a new lens [51]. And in zebrafish, at least three members of the BMP family have been associated with fin regeneration [52]. Inhibition of BMP activity using the BMP antagonist, noggin, caused a reduction in blastema cell proliferation and a reduction in bone matrix deposition. Similarly, in experiments using transgenic Xenopus tadpole with increase noggin expression, both tail and limb regeneration were inhibited [53]. Finally, BMP has been shown to be essential for the correct establishment of the dorso-ventral axis during planarian regeneration [54,55]. BMPs have also been associated with normal gastrointestinal tract development in vertebrates, and alterations in their expression caused morphological abnormalities in the intestinal tissues ([56,57]). BMP have also been shown to be required for proper cloaca formation [58]. Therefore, the overexpression of BMP during intestinal regeneration observed in our system is consistent with its role in the proper formation of the digestive tract organs.

### • How do microarray results correlate with cellular processes that occur during intestinal regeneration?

In order to understand how changes in gene expression are associated with changes at the tissue and organ levels it is important to have a clear understanding of the cellular events that underlie the regenerative process. For this reason, our laboratory has described the intestinal regeneration process in *H. glaberrima *from the cellular perspective [6-12]. Two events in particular are to be highlighted in view of their possible association with the differential gene expression profile, one is the change in cytoskeletal proteins and the second the remodeling of the extracellular matrix.

#### Cytoskeletal Proteins

Previous studies from our laboratory have shown dramatic changes in the cytoskeleton of cells in the regenerating intestine. Many of these changes were associated with de-differentiation of the mesenterial muscle during the initial regeneration stages and with the processes of myogenesis, innervation and luminal epithelial formation in later regenerative stages [6,8,10,11]. In fact, during the regenerative process muscle cells actively extrude their contractile apparatus as spindle-like structures (SLSs) [8]. This has also been shown in other echinoderm regenerative phenomena [1,59]. We can speculate that the under-expression of some actin and myosin isoforms is associated with this dedifferentiation process. Similarly, the over expression of some actin isoforms could be associated with the migration of cells into the regenerating tissue [7,9], or with the eventual differentiation of cells from committed precursors [11]. Nonetheless, these hypotheses will need to be verified in future experiments using *in situ *hybridization and immunochemical analyses.

One of the most interesting findings is the under expression of gelsolin in early regenerative stages. Results by Cowin and colleagues [60] have shown that gelsolin mRNA was upregulated and gelsolin protein was associated with actin filaments in the fetal skin of embryonic day 19 rats, but not at embryonic day 17. This is the age gap when fetal wounds properties change from being scar-free to scar-forming. In culture, skin from 17 day embryonic rats can epithelialize an excisional wound in culture, but this ability is lost by embryonic day 19. Thus, our finding of a correlation between gelsolin gene under-expression and early intestinal regeneration suggests that gelsolin might play an important inhibitory role in the process of wound healing and regeneration.

#### ECM

One of the events that is common to most regenerative processes is the remodeling of the extracellular matrix. These changes, which have been well studied particularly during epithelial wound healing, involve the replacement of the ECM by a transient ECM that facilitates the cellular events associated with the regenerative response [61]. In the holothurian intestinal system we have previously shown a dramatic extracellular remodeling during the early stages of regeneration [12]. During this time collagen and laminin immunoreactivities disappear from the mesentery that gives rise to the new organ. Similarly there is a rise in the activity of MMPs that coincides with the major changes in the ECM. Moreover, we showed that inhibition of MMPs at this time point causes an inhibition of the regeneration process [12]. At the microscopic level, the changes of ECM and MMP activity coincide with an increase presence of phagocytic amoebocytes in the regenerating areas [8]. Therefore, the differential expression of various molecules associated with ECM remodeling, particularly the MMPs provide specific targets to define their role in the regeneration process.

### • How do our results serve to advance the regeneration field?

The cellular and molecular processes of organ regeneration have received increasing attention in recent years [5]. In fact, the question of **what controls organ regeneration? **has been highlighted as one of the top scientific issues that deserves to be studied with high priority [62]. However, organ regeneration remains one of the least understood biological processes, particularly at the molecular level. Progress toward characterizing the molecular basis of regenerative processes has been modest for at least three reasons [63,64]. **First**, the regenerative capacity of most vertebrate animals is relatively limited [3], so popular biomedical models are not very useful. For example, some mammalian tissues (*e.g*., intestinal epithelia) and organs (*e.g*., skin and liver) can readily regenerate, but others, like heart and nervous tissues, have little or no regenerative capacity. **Second**, although development is a process closely akin to regeneration, most animals used extensively to study the molecular aspects of development (worms, fruitflies, zebrafish, chickens and mice) lack robust regenerative capacities. **Third**, those animals with remarkable regenerative capacities, such as coelenterates, flatworms, tunicates, newts and echinoderms have been little studied at the molecular/genetic level [1,20,65].

This has started to change and ongoing projects promise a bright future for the identification of regeneration-associated genes. Among the studies/models that need to be highlighted in terms of EST/genomic are those in amphibians [30,66,67], zebrafish [27,68,69], ascidians [70,71] planaria [4,55,72-77] and Hydra [78-81]. However, the only echinoderm studies are those of the sea urchin genome [82], which ironically is the echinoderm group with the least regenerative capacities [83].

Analyses of regeneration-associated genes in multiple models will lead to important comparative studies among species with highly developed regenerative capacities or between closely related species where good and poor regenerators are found. The importance of comparative studies using a large range of animal models was highlighted by Sanchez Alvarado and Tsonis [4] who described the differences and similarities in the regeneration mechanisms among diverse animal species. Other investigators pointed out that the echinoderms were largely excluded from this analysis [84], a fact explained by one of the authors [21] as being due to the lack of modern molecular tools to study the regeneration phenomena. Nonetheless, all investigators agreed that the application of genome-wide profiling approaches (as well as other tools) to the echinoderms would provide significant contributions to the understanding of regeneration. Thus, the present results represent an important step toward the molecular study of the amazing regenerative capacities of echinoderms and more specifically toward their use to better understand how organs can be regenerated.

## Conclusion

Despite the apparent simplicity of the echinoderms, here we show that the organ regeneration is a complex process that involves the up-regulation and down-regulation of thousands of genes. Consistent with previous findings, these genes have been reported as involved in regeneration, development, ECM rearrangement, cytoskeleton reorganization and wound healing such as Wnt-9, BMP-1, Hox12, Tubulin, Tens-R, MMPs and gelsolin respectively. Moreover, a large number of unknown genes were also found to be differentially expressed at different stages of regeneration and might represent novel genes. Finally, here we show that intestine regeneration in the sea cucumber is a novel and important model for studies to identify and characterize the molecular basis of regenerative processes.

## Methods

### Microchip preparation

We have used the Agilent platform to design, perform and analyze custom-made *H. glaberrima *arrays. For this we used the "eArray" server from Agilent to design 60-mer probes that were synthesized on the microarray. A design with 15744 spots on each array and 8 arrays per slide was selected. All sequences from the cDNA libraries of normal and regenerating intestines were represented at least twice. Thus, 14352 (91.2%) probes were from 6287 clean sequences from *H. glaberrima*. We also spotted other sequences. These sequences represented genes associated to development, regeneration, proliferation, wound healing and growth. This pool included 150 probes from the sea cucumber *Apostichopus japonicus *(reported by Zheng et al. 2006), 8 from other holothurians that could be found in the NCBI database and 658 from the sea urchin *S. purputatus *(whose genome was recently sequenced [82]). Finally, 40 probes were from vertebrate organisms including mice, zebrafish and axolotl. Technical controls were 536 (3.4%).

#### Animals and treatments

Adult sea cucumbers (10~12 cm long) were collected from the north-eastern rocky shores of Puerto Rico. Animals were eviscerated at day 0, by intra-coelomic injection of 0.35 M KCl. They were left undisturbed in the aquaria to allow regeneration for 3, 7 and 14 days. At each time-point, animals were anesthetized by immersion in ice-cold sea water for 45 min. A dorsal incision was made to completely expose the internal cavity and allow the dissection of the intestinal primordium (3-, 7- or 14-dpe). After dissection, the regenerating intestines were placed in RNAlater^® ^(Applied biosystems/Ambion, Austin TX) solution for RNA extraction. Uneviscerated (normal) animals were used as controls. One group was injected once with 1 mg lipopolysaccharides (LPS) diluted in 0.5 mL filtered sea water as reported by [85]. Intestines of the LPS treated animals were dissected 48 hrs after injection.

### RNA Extraction

RNA was extracted using a combination of the [86] method using Tri-reagent^® ^(N.93289, Sigma, St Louis, MO) and the RNAeasy mini kit from Quiagen (Valencia, CA) The concentration and integrity of RNA were determined using the NanoDrop-1000 Spectrophotometer (NanoDrop Technologies, Rockland, DE) and the 2100 Bioanalyzer (Agilent) with an RNA 6000 Nano LabChip^® ^Kit at the Functional Genomics Research center (FGRc -UPR, Puerto Rico).

### Synthesis and labeling of cRNA

RNA samples were amplified and labeled using the Low Input Fluorescent Linear Amplification kit (Agilent). Briefly, cDNA was first synthesized combining 300 ng of RNA with T7 promoter primers, 5× First Strand Buffer, 0.1 M DTT, 10 mM dNTP mix, MMLV RT and RNaseOUT. This reaction mix was incubated at 40°C for 2 h. Then, cRNA synthesis and dye incorporation were performed as follows: cDNA of the first reaction was combined with cyanine-3-CTP (Cy3, 10 mM) or cyanine 5-CTP (Cy5,10 mM) and the Transcription Master Mix (4× transcription buffer, 0.1 M DTT, NTP mix, 50% PEG, RNAseOUT, inorganic pyrophosphase and T7 RNA polymerase) at 40°C for 2 h. The fluorescently labeled cRNA products were purified using RNeasy mini spin columns (QIAGEN), subsequently analyzed for yield and dye incorporation using the NanoDrop, and finally stored at -80°C until needed. Only samples that presented a concentration of more than 8 pmol/mg (measurement representing efficiency of dye incorporation) were used for hybridization as recommended by the manufacturer.

### Hybridization & Scanning

Cy3 and Cy5-labeled cRNAs were combined, fragmented, and hybridized to an 8 × 15K 60-mer oligo microarray (Agilent) for 17.5 h at 60°C with continuing rotation at 4 rpm. After hybridization, slide arrays were subjected to two successive washes (wash solution 1: 6× SSPE, 0.005% N-Lauroylsarcosine, and wash solution 2: 0.06× SSPE, 0.005% N-Lauroylsarcosine) and dried with the Stabilization and Drying Solution (Agilent). Arrays were immediately scanned in a G250B Microarray Scanner (Agilent) to obtain fluorescence intensities and Cy5/Cy3 ratios for each gene on the array (see below). Scanning parameters were used as recommended by the manufacturer. The resolution settings for scanning were 5 μm using minimum (10%) and maximum (100%) Photo Multiplier Tube detection sensitivities.

### Microarray Experimental Design

**Figure 8:**
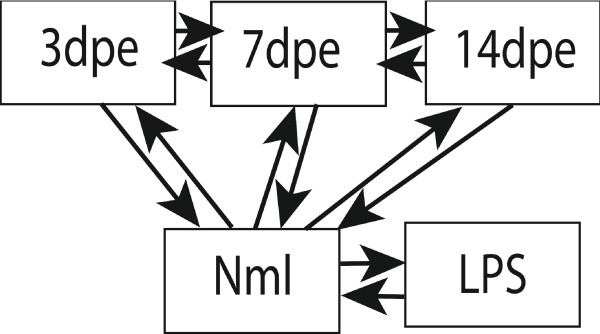
**Microarray experimental design**. Normal (Nml) and regenerating intestines at 3, 7 and 14 dpe (days post-evisceration) were compared among them. Each experiment was replicated as a dye swap with different biological samples. LPS treated tissues where used to normalize the expression for immune activated genes. Arrows show direct comparisons.

The experimental design consisted of comparisons between Normal (non-eviscerated) intestinal tissues and 3-, 7-, and 14-dpe regenerating intestines. To determine differences in gene expression between regenerating stages we also compared directly 3- vs. 7-dpe and 7- vs. 14-dpe (Figure 8). Comparisons were also made between the intestinal tissues of normal and immune activated animals (injected LPS). There were only 48 genes differentially expressed between normal and LPS-immune activated intestines and these genes will be discussed elsewhere. (F. Ramirez-Gomez et al. in prep.) These genes were subtracted from the regeneration profile to ensure that genes associated with the regeneration phenomenon were not differentially expressed due any possible immune activation (e.g., because of bacterial invasion during the evisceration process).

Technically, for each comparison, the microarray experiment was done twice, one using different biological samples, each consisting of a sample pool from 3 animals. Of the two biological replicates one was always performed as a dye-swap in order to correct any dye bias.

### Microarray data analysis

Microarray data was extracted with Agilent's Feature Extraction software (version 5.1) and analyzed using the Limma package of Bioconductor. The data discussed in this publication have been deposited in NCBI's Gene Expression Omnibus [87] and are accessible through GEO Series accession number GSE16182 . The analysis of the microarray data consisted of the following steps: 1) within-array and between-array normalizations; 2) fitting the data to a linear model; and 3) computing differential gene expression. For normalization purposes [88] MA-plots were generated representing the (R, G) data (R = red for Cy5 and G = green for Cy3), in which the log ratio of R versus G (M value = log_2 _R/G) was plotted against the overall intensity of each spot (A value = log_2 _(R + G)/2. Within-array normalization was first applied and M-values were normalized within each array using the Global Loess Normalization method. Aquantile normalization was then applied to the A-values as a method for between-array normalization, to assure that the intensities and log-ratios had similar distributions across arrays. To estimate the average M-value for each gene and assess differential gene expression, a simple linear model was fit to the data, and M-value averages and standard deviations for each gene were obtained. To find genes with significant expression changes between groups, empirical Bayes statistics were applied to the data by moderating the standard errors of the estimated M-values. P-values were obtained from the moderated t-statistic and corrected for multiple testing with the [89] method. The null hypothesis, that there is no differential expression of genes between regeneration stages compared with normal tissues, was rejected for p-values lower than 0.05. Thus, the change in expression is given by the fold change while the believability of the change is given by the odds. Alternative to this method, the GenePix Pro 6.0.1.26 and Acuity 4.0.0.60 software (from Molecular Devices, Sunnyvale CA) was also used to analyze gene expression values (log ratios) and perform clustering analyses. Methods used to visualize the expression profiles included hierarchical [90] and non-hierarchical clustering such as Self-Organizing Maps (SOM, [91]).

### Gene expression analysis

The differential expression of the genes of interest was semi-quantitatively determined by gene-specific relative RT-PCR. Primers were designed for optimal performance using the Primer3 and Net primer web-servers [see additional file 4 for the list of primer sequences]. All primers were synthesized by AlphaDNA (Montreal, Canada). PCRs were performed in the Mastercycler ep (Eppendorf, Westbury, NY) using the Promega's Taq-Polimerase kit (Madison, USA). Cycling conditions for the amplified products were as follow: 94°C × 1 min, 55°C (± 3°C) × 1 min, 72°C × 1 min; all performed for 35~40 cycles. Optical densities of the PCR products were measured using Photoshop (Adobe). Values were extracted for background and then normalized against NADH-dehydrogenase unit 5 (NADH, 241 bp). Each stage of regeneration was analyzed with at least an N = 7, each sample represented the intestine of one individual. Statistical test of mean comparison was carried in R  under the null hypothesis that there is no differential expression between different stages of regeneration and normal tissues. Significance of the paired t-test was assumed at p < 0.05.

## List of abbreviations

**cDNA**: complementary DNA; **EST**: Expressed sequence tag; **Dpe**: Days post evisceration; **RT-PCR**: reverse transcriptase polymerase chain reaction; **LPS**: lipopolysaccharides; **MMP**: Matrix metalloproteases; **GO**: Gene Ontology; **ORF**: Open Reading Frame; **UTR**: Untranslated region; **mRNA**: Messenger RNA; **SLS**: spindle like structure; **ECM**: Extra-Cellular Matrix.

## Authors' contributions

POP designed and carried out the study, analyzed and interpreted the data, and drafted the manuscript. FRG helped in the design and participated in the molecular experiments. CRC, JRL, ESC, ATMM and KT participated in cDNA library construction, EST characterization and analysis and gene identification and sequencing. JPO, SGD, FSD, JH, and CDA performed most of the validation experiments and participated in the analysis of data. HOZ helped in bioinformatics analysis. JEGA conceived the study, participated in its design, data analyses and helped in the manuscript preparation. All authors have given final approval of the version to be published.

## Supplementary Material

Additional file 1 **Microarray internal controls performance**. Numerical and graphical representation of the internal control signals. Click here for file

Additional file 2 **Normalization procedure**. Example of two random selected arrays before and after normalization. Click here for file

Additional file 3 **Dye-swap hybridization**. Bottom left corner of one array and the same corner in the corresponding dye-swap microarray Click here for file

Additional file 4 **Primers**. List of primers used for validation. Click here for file
