# Supplementary material for: Gene expression profiling of intestinal regeneration in the sea cucumber
Source: BMC Genomics. 2009 Jun 8;10:262. doi: 10.1186/1471-2164-10-262 (PMC2711116; doi:10.1186/1471-2164-10-262)
Supplement: Additional file 4 — Primers. List of primers used for validation. [file 1471-2164-10-262-S4.pdf]

Additional File 4: Primers used for validation.

Gene ID	Dir	Primer	Prod. Size
MMP-11	Fwd	TGATGAAGACGGGAATAATG	487
	Rev	AGAGGACAACCAAGCACGA	
MMP-14	Fwd	TGCTTGGCATTCAGACACTT	357
	Rev	ATCCTGGGAGAGGGTTTAGG	
MMP-15	Fwd	CGGTTGTTCATCGTATGTTTG	366
	Rev	CATTCCTCCTGAGTGGGTGTA	
Tens-R	Fwd	GCAAACATACCACAACGAGA	236
	Rev	CACCCCGTACAAATCCAAG	
Wnt	Fwd	AAAAGGCGTCACTCTGCT	357
	Rev	CACACACCCCAAAATCG	
TCTP	Fwd	TTTGCTATTTCCCAGGAT	288
	Rev	TTCATTTCTGTAAGCCCAT	
BMP-1	Fwd	TACAATAAACAGAAAGGGCT	343
	Rev	TGGCAACAAGTGGGGTA	
Cadh-1	Fwd	CATGACAGGGAAGGTAGGAGA	385
	Rev	CTGAGATGAGGATGAACGCA	
Unk-E	Fwd	AACTCAGGCGTGGAGATAGAAC	258
	Rev	ACACAAGGAGGAATTTAGAAGGG	
Unk-F	Fwd	TATTTTCCAGTGCCAGATTCAGT	487
	Rev	GAGTTCTACAAAGCTCACCCAGA	
Unk-G	Fwd	GAAGCCTTTTGTCCTTACGA	348
	Rev	TGTTTGTGGTTGGATAGTGC	
U-5501	Fwd	AAACACAGGAGGTTGGGGA	400
	Rev	ACGCTGGTGGCTTTCTTG	
U-5242	Fwd	GCAGTAAGAGACAGTAACGAAACA	294
	Rev	AGAAATAGAAAGACTCCCACCAC	
U-4874	Fwd	AAATGGCCCTCCCTACCTC	482
	Rev	TATGCTTCCTCACCCAATCC	
NADH	Fwd	CGGCTACTTCTGCGTTCTTC	241
	Rev	ATAGGCGCTGTCTCACTGGT	
Actin -1	Fwd	ATCGTGTTGCATTCGTGTTG	200
	Rev	TCCTTCAGGTGGTCAGTCCT	
Actin -2	Fwd	AAGCCACCGTCAGTGAAAAA	399
	Rev	CCGTGGACAAGATGACAAGA	
Actin -3	Fwd	CTCACTTCGTTGGAACCACA	156
	Rev	TGCATCTTCATAGCCAGCTT	
HgHox12	Fwd	TGCGGAGTACAGGGGTTTAC	191
	Rev	GACTTGCAGACGAGGACACA	
